# Supplementary material for: Functional Alteration in the Brain Due to Tumour Invasion in Paediatric Patients: A Systematic Review
Source: Cancers (Basel). 2023 Apr 6;15(7):2168. doi: 10.3390/cancers15072168 (PMC10093754; doi:10.3390/cancers15072168)
Supplement: Supplementary file 1 [file cancers-15-02168-s001.zip › cancers-2255486-supplementary.pdf]

SUPPLEMENTARY MATERIALS

Table S1: PRISMA 2020 Checklist

Section and Topic	Item #	Checklist item	Location where item is reported (page no)
**TITLE**			
Title	1	Identify the report as a systematic review	1
		ABSTRACT	
Abstract	2	See the PRISMA 2020 for Abstract checklist	S2
**INTRODUCTION**			
Rationale	3	Describe the rationale for the review in the context of existing knowledge	2
Objectives	4	Provide an explicit statement of the objective(s) or question(s) the review addresses	3
**METHODS**			
Eligibility criteria	5	Specify the inclusion and exclusion criteria for the review and how studies were grouped for the syntheses	3
Information sources	6	Specify all databases, registers, websites, organisations, reference lists and other sources searched or consulted to identify studies. Specify the date when each source was last searched or consulted	4, Figure 1
Search strategy	7	Present the full search strategies for all databases, registers and websites, including any filters and limits used	Figure 1, S3
Selection process	8	Specify the methods used to decide whether a study met the inclusion criteria of the review, including how many reviewers screened each record and each report retrieved, whether they worked independently, and if applicable, details of automation tools used in the process	4, 5, 6
Data collection process	9	Specify the methods used to collect data from reports, including how many reviewers collected data from each report, whether they worked independently, any processes for obtaining or confirming data from study investigators, and if applicable, details or automation tools used in the process	NA
Data items	10a	List and define all outcomes for which data were sought. Specify whether all results that were compatible with each outcome domain in each study were sought (e.g. for all measures, time points, analyses), and if not, the	7, 8, 9, 10, 11, 12

Section and Topic	Item #	Checklist item	Location where item is reported (page no)
		methods used to decide which results to collect	
	10b	List and define all other variables for which data were sought (e.g. participant and intervention characteristics, funding sources). Describe any assumptions made about any missing or unclear information	Table 2, 3, 4
Study risk of bias assessment	11	Specify the methods used to assess risk of bias in the included studies, including details of the tool(s) used, how many reviewers assessed each study and whether they worked independently, and if applicable, details of automation tools used in the process	7
Effect measures	12	Specify for each outcome the effect measure(s) (e.g. risk ratio, mean difference) used in the synthesis or presentation of results	Table 2, page 13
Synthesis methods	13a	Describe the processes used to decide which studies were eligible for each synthesis (e.g. tabulating the study intervention characteristics and comparing against the planned groups for each synthesis (item #5))	Table 2,3,4
	13b	Describe any methods required to prepare the data for presentation or synthesis, such as handling of missing summary statistics, or data conversions	7, 8
	13c	Describe any methods used to tabulate or visually display results of individual studies and syntheses	8
	13d	Describe any methods used to synthesize results and provide a rationale for the choice(s). If meta-analysis was performed, describe the model(s), method(s) to identify the presence and extent of statistical heterogeneity, and software package(s) used	NA
	13e	Describe any methods used to explore possible causes of heterogeneity among study results (e.g. subgroup analysis, meta-regression).	NA
	13f	Describe any sensitivity analyses conducted to assess the robustness of the synthesized results	5, 8, 9
Reporting bias assessment	14	Describe any methods used to assess risk of bias due to missing results in a synthesis (arising from reporting biases)	NA
Certainty assessment	15	Describe any methods used to assess certainty (or confidence) in the body of evidence for an outcome	NA
**RESULTS**			
Study selection	16a	Describe the results of the search and selection process, from the number of	Figure 1

Section and Topic	Item #	Checklist item	Location where item is reported (page no)
		records identified in the search to the number of studies included in the review, ideally using a flow diagram	
	16b	Cite studies that might appear to meet the inclusion criteria, but which were excluded, and explain why they were excluded	Figure 1
Study characteristics	17	Cite each included study and present its characteristics	Table 2,3,4
Risk of bias in studies	18	Present assessments of risk of bias for each included study	NA
Results of individual studies	19	For all outcomes, present, for each study: (a) summary statistics for each group (where appropriate) and (b) an effect estimate and its precision (e.g. confidence/credible interval), ideally using structured tables or plots	Table 2, 3
Results of syntheses	20a	For each synthesis, briefly summarise the characteristics and risk of bias among contributing studies.	NA
	20b	Present results of all statistical syntheses conducted. If meta-analysis was done, present for each the summary estimate and its precision (e.g. confidence/credible interval) and measures of statistical heterogeneity. If comparing groups, describe the direction of the effect	Table 4, page 9
	20c	Present results of all investigations of possible causes of heterogeneity among study results	12
	20d	Present results of all sensitivity analyses conducted to assess the robustness of the synthesized results	Table 4, 5
Reporting biases	21	Present assessments of risk of bias due to missing results (arising from reporting biases) for each synthesis assessed	NA
Certainty of evidence	22	Present assessments of certainty (or confidence) in the body of evidence for each outcome assessed	Page 12, Table 4
**DISCUSSION**			
Discussion	23a	Provide a general interpretation of the results in the context of other evidence	26 - 30
	23b	Discuss any limitations of the evidence included in the review	30
	23c	Discuss any limitations of the review processes used	30
	23d	Discuss implications of the results for practice, policy, and future research	30

Section and Topic	Item #	Checklist item	Location where item is reported (page no)
**OTHER INFORMATION**			
Registration and protocol	24a	Provide registration information for the review, including register name and registration number, or state that the review was not registered	NA
	24b	Indicate where the review protocol can be accessed, or state that a protocol was not prepared	NA
	24c	Describe and explain any amendments to information provided at registration or in the protocol	NA
Support	25	Describe sources of financial or non-financial support for the review, and the role of the funders or sponsors in the review	NA
Competing interests	26	Declare any competing interests of review authors	NA
Availability of data, code and other materials	27	Report which of the following are publicly available and where they can be found: template data collection forms; data extracted from included studies; data used for all analyses; analytic code; any other materials used in the review	4

[Ref] Page, M.J.; McKenzie, J.E.; Bossuyt, P.M.; Boutron, I.; Hoffmann, T.C.; Mulrow, C.D.; The PRISMA 2020 statement: an updated guideline for reporting systematic reviews. BMJ 2021;372:n71. doi: 10.1136/bmj.n71

Table S2: PRISMA 2020 for Abstract Checklist

Section and Topic	Item #	Checklist item	Reported (Yes/No)
**TITLE**			
Title	1	Identify the report as a systematic review	Yes
**BACKGROUND**			
Objectives	2	Provide an explicit statement of the main objective(s) or question(s) the review addresses	Yes
**METHODS**			
Eligibility criteria	3	Specify the inclusion and exclusion criteria for the review	No
Information sources	4	Specify the information sources (e.g. databases, registers) used to identify studies and the date when each was last searched	Yes
Risk of bias	5	Specify the methods used to assess risk of bias in the included studies	No
Synthesis of results	6	Specify the methods used to present and synthesise results	Yes
**RESULTS**			
Included studies	7	Give the total number of included studies and participants and summarise relevant characteristics of studies	Yes
Synthesis of results	8	Present results for main outcomes, preferably indicating the number of included studies and participants for each. If meta-analysis was done, report the summary estimate and confidence/credible interval. If comparing groups, indicate the direction of the effect (i.e. which group is favoured)	Yes
**DISCUSSION**			
Limitations of evidence	9	Provide a brief summary of the limitations of the evidence included in the review (e.g. study risk of bias, inconsistency and imprecision)	Yes
Interpretation	10	Provide a general interpretation of the results and important implications	Yes

Section and Topic	Item #	Checklist item	Reported (Yes/No)
**OTHER**			
Funding	11	Specify the primary source of funding for the review	No (ND)
Registration	12	Provide the register name and registration number	No (ND)

[Ref] Page, M.J.; McKenzie, J.E.; Bossuyt, P.M., Boutron, I.; Hoffmann, T.C.; Mulrow, C.D.; The PRISMA 2020 statement: an updated guideline for reporting systematic reviews. BMJ 2021;372:n71. doi: 10.1136/bmj.n71

Table S3: Advanced Search Keyword chain in PubMed

1	PubMed	((((((children) OR (child)) OR (paediatrics)) OR (minor)) OR (junior)) AND (((brain tumor) OR (brain tumour)) OR (brain neoplasm)) OR (brain cancer))) AND (((fMRI) OR (functional MRI)) OR (functional magnetic resonance imaging))

Table S4: Quality Assessment of Selected Studies by NHLBI, NIH

S4.1 Quality assessment of case-controlled study

Y; Yes, N; No, Other (CD; cannot determine, NA; not applicable, NR; not reported)

	Criteria	Riva et al.	Robinson et al.	Hoang et al.	Zou et al.	Cheng et al.
1	Was the research question or objective in this paper clearly stated and appropriate?	Y	Y	Y	Y	Y
2	Was the study population clearly specified and defined?	Y	Y	Y	Y	Y
3	Did the authors include a sample size justification?	N	N	N	Y	Y
4	Were controls selected or recruited from the same or similar population that gave rise to the cases (including the same timeframe)?	Y	Y	Y	Y	Y
5	Were the definitions, inclusion and exclusion criteria, algorithms or processes used to identify or select cases and controls valid, reliable, and implemented consistently across all study participants?	Y	Y	Y	Y	Y
6	Were the cases clearly defined and differentiated from controls?	Y	Y	Y	Y	Y
7	If less than 100 percent of eligible cases and/or controls were selected for the study, were the cases and/or controls randomly selected from those eligible?	N	N	N	CD	N
8	Was there use of concurrent controls?	N	N	N	N	N
9	Were the investigators able to confirm that the exposure/risk occurred prior to the development of the condition or event that defined a participant as a case?	Y	Y	Y	Y	Y
10	Were the measures of exposure/risk clearly defined, valid, reliable, and implemented consistently (including the same time period) across all study participants?	Y	Y	Y	Y	Y
11	Were the assessors of exposure/risk blinded to the case or control status of participants?	N	N	N	N	N
12	Were key potential confounding variables measured and adjusted statistically in the analyses? If matching was used, did the investigators account for matching during study analysis?	Y	Y	Y	Y	Y

S4.2 Quality Assessment tool for Before - After (Per-Post) Studies With No Control Group

No	Criteria	Talabaev et al.	He et al.	Delion et al.	Zhu et al. (2017)	Boerwinkle et al.	Zhu et al. (2019)	Anwar et al.	Roland et al.	Lorenzen et al.	Wolfe et al.	Li et al.
1	Was the study question or objective clearly stated?	Y	Y	Y	Y	Y	Y	Y	Y	Y	Y	
	Were eligibility/selection criteria for the study population prespecified and clearly described?	Y	Y	Y	Y	Y	Y	Y	Y	Y	Y	Y
3	Were the participants in the study representative of those who would be eligible for the test/service/intervention in the general or clinical population of interest?	Y	Y	Y	Y	Y	Y	Y	Y	Y	Y	Y
4	Were all eligible participants that met the prespecified entry criteria enrolled?	Y	Y	Y	Y	Y	Y	Y	Y	Y	Y	Y
5	Was the sample size sufficiently large to provide confidence in the findings?	N	Y	N	Y	Y	N	Y	Y	Y	N	N
6	Was the test/service/intervention clearly described and delivered consistently across the study population?	N	Y	N	Y	N	Y	N	N	Y	Y	N
7	Were the outcome measures prespecified, clearly defined, valid, reliable, and assessed consistently across all study participants?	Y	Y	Y	Y	Y	Y	N	Y	Y	Y	Y
8	Were the people assessing the outcomes blinded to the participants' exposures/interventions?	N	N	N	N	N	N	N	N	N	N	N
9	Was the loss to follow-up after baseline 20% or less? Were those lost to follow-up accounted for in the analysis?	Y	Y	Y	Y	Y	Y	Y	Y	Y	Y	Y

10	Did the statistical methods examine changes in outcome measures from before to after the intervention? Were statistical tests done that provided p values for the pre-to-post changes?	Y	Y	Y	Y	Y	Y	Y	Y	Y	Y	Y
11	Were outcome measures of interest taken multiple times before the intervention and multiple times after the intervention (i.e., did they use an interrupted time-series design)?	N	Y	Y	Y	N	Y	N	N	Y	N	Y
12	If the intervention was conducted at a group level (e.g., a whole hospital, a community, etc.) did the statistical analysis take into account the use of individual-level data to determine effects at the group level?	Y	Y	Y	Y	Y	Y	Y	Y	Y	Y	Y

Y; Yes, N; No, Other (CD; cannot determine, NA; not applicable, NR; not reported)
